# Safe and supported: Mitigating psychotic experiences in Irish adolescents – A population-based study

**DOI:** 10.1017/S0033291726104632

**Published:** 2026-05-11

**Authors:** John Hoey, Tomasz Szank, Jessica Ohland, Ronan Fleury, Lorna Staines, Niamh Dooley, Emmet Power, Ian Kelleher, David Cotter, Mary Cannon

**Affiliations:** 1 Department of Psychiatry, Royal College of Surgeons in Ireland School of Medicine: RCSI School of Medicine, Dublin, Ireland; 2 Faculty of Science and Health, Technological University of the Shannon Midlands Midwest - Athlone Campus, Ireland; 3 Department of Psychiatry, Royal College of Surgeons in Ireland Faculty of Medicine and Health Sciences, Ireland; 4 Centre for Clinical Brain Sciences, Division of Psychiatry, The University of Edinburgh Neuroscience, Edinburgh, UK

**Keywords:** adolescent, emotions, gender identity, health equity, mental health, minority groups, psychotic disorders, psychotic experiences, social support

## Abstract

**Background:**

Psychotic experiences (PEs) in are associated with elevated risk for mental health difficulties. This study examined predictors of PEs, inclusive of the role of gender, ethnicity, and protective factors.

**Methods:**

Data were drawn from a 2021 Planet Youth survey of adolescents (n = 4,005). PEs were measured using the adolescent psychotic symptom screener. Effects of psychosocial predictors on PEs were measured by fitting multivariable logistic regression main effect and joint exposure models.

**Results:**

29.8% reported PEs. Black/Asian/Other minorities had elevated odds (a*OR* = 1.59, 95% CI 1.26–2.02, *p* < .001). Increased odds in males, females, undisclosed gender and non-binary/transgender with elevated emotional/behavioural difficulties (a*OR* = 4.47, 95% CI 3.53–5.67, *p* < .001; a*OR* = 3.25, 95% CI 2.59–4.08, *p* < .001; a*OR* = 4.83, 95% CI 2.58–9.02, p < .001; a*OR* = 4.33, 95% CI 2.69–6.97, *p* < .001 respectively). High odds in undisclosed gender with low emotional/behavioural difficulties (a*OR* = 4.36, 95% CI 1.50–12.66, *p* = .007). Lower odds from perceived school/home safety (a*OR* = 0.69, 95% CI 0.58–0.83, *p* < .001 and (a*OR* = 0.81, 95% CI 0.66–0.99, *p* = .038, respectively). Elevated odds from recent adversities (a*OR* = 1.91, 95% CI 1.47–2.49, *p* = .011) attenuated by parental support (a*OR* = 1.76, 95% CI 1.17–2.65, *p* < .001). Each additional adversity (>12 months) increased odds (a*OR* = 1.12, 95% CI 1.07–1.17, *p* < .001).

**Conclusions:**

Findings highlight the interplay of risk and protective factors in adolescent PEs, with increased vulnerability among minoritized youth. Results support targeted interventions to reduce mental health disparities.

## Introduction

Psychotic experiences (PEs), encompassing disturbances of perception and thought such as hallucinations and delusions, are relatively common during adolescence, with an estimated prevalence of 7.5% in general population studies (Kelleher et al., 2012). These experiences, which may involve hearing voices that others cannot hear or holding unusual beliefs such as feeling under the control of special powers, often occur below the threshold of clinical psychotic disorders. However, they are significant as they predict an increased likelihood of developing psychotic disorders later in life and are associated with a range of broader mental health challenges, including anxiety, depression, and suicidal ideation (Healy et al., 2018; Kelleher & Cannon, 2012; Staines et al., 2023). PEs are also linked to poorer long-term developmental outcomes, highlighting adolescence as a critical period for understanding mental health vulnerabilities (Staines et al., 2023).

Minority stress theory posits that unique, additive stressors, including discrimination, victimization, expectations of rejection, and internalized stigma, which add to general life stress and increase vulnerability to mental health difficulties, exacerbate the risk of mental health difficulties among individuals in stigmatized groups (Meyer, Russell, Hammack, Frost, & Wilson, 2021). Adverse experiences and stigma-related stress may disrupt emotional regulation and social functioning, contributing to an increased prevalence of PEs in these populations (Dolphin, Dooley, & Fitzgerald, 2015; Flentje, Heck, Brennan, & Meyer, 2020). Studies have identified adverse life events and victimization, as key risk factors, while protective influences, including strong parental support, neighborhood social cohesion, and engagement in physical activity have been shown to mitigate these risks (Crush et al., 2018; Dolphin et al., 2015). Evidence further suggests that females report higher rates of PEs than males, although social stressors appear to have a stronger impact on males (Stainton et al., 2021), with marginalized groups, such as sexual, gender, and ethnic minorities, experiencing disproportionately high exposure to stigma-related stressors, including bullying, violence, and socioeconomic hardship, due to their stigmatized social status (Meyer, 2003; Vargas et al., 2020).

Meyer’s protective factors, such as parental warmth and acceptance, however, can reduce the likelihood of PEs. They have been associated with greater emotional stability, life satisfaction, and academic success (Mendo-Lázaro, León-Del-Barco, Polo-Del-Río, Yuste-Tosina, & López-Ramos, 2019; Meyrose et al., 2018). Similarly, fostering supportive environments for adolescents has been shown to lower PEs (Brokmeier et al., 2020; Hanevik, Hestad, Lien, Teglbjaerg, & Danbolt, 2013).

This study examines psychosocial predictors of PEs in adolescent through the lens of Minority Stress and focusing on the role of risk and protective factors. It explores the impact of adverse life events, financial strain, perceived safety in home and school settings, and parental and school-based support. It also examines whether observed psychosocial risk patterns among sexual, gender, and ethnic minority youth are consistent with processes described in minority stress theory. The goal is to inform targeted interventions that reduce stigma, support adolescent resilience, and promote mental health equity.

## Methods

### Study design

This study involved secondary analysis of the largest school-based survey of adolescents from three regions in Ireland: Cavan, Monaghan, and North Dublin carried out in October–November 2021 as part of the PY, an evidence-based prevention program originally developed in Iceland and adapted for use in Ireland to examine factors influencing adolescent well-being through cross-sectional surveys distributed in schools and equivalent YouthReach settings participating in the initiative. The target population were students in second to fifth year of secondary school (age range: 13–19; median age 15–16) and equivalent adolescents from YouthReach centers. A total of 40 schools participated across three regions. 100% of the invited educational facilities in the Cavan (10 post-primary schools and two YouthReach centers) and Monaghan (11 post-primary schools and two YouthReach centers) region participated with the expected student cohort of 1,942 adolescents. 75% of the invited centers from North Dublin participated. Surveys were administered in classroom settings using iPads or PCs, with students completing self-administered questionnaires. The design ensured participant anonymity, with no identifiable personal or school-level data included in the dataset. Data collection was conducted by trained local teams, and procedures followed the standard PY implementation protocol.

### Ethics

Ethical approval for this secondary analysis was obtained from the Research Ethics Committee of the Royal College of Physicians in Ireland (RECSAF 144). All procedures adhered to principles of confidentiality, research integrity, and the ethical use of secondary data.

### Measures

#### Outcome variable

PEs as the outcome variable were assessed using the 7-item APSS (Kelleher, Harley, Murtagh, & Cannon, 2011). This instrument demonstrated good predictive power for PEs with the question ‘Have you ever heard voices or sounds that no-one else can hear?’ being the strongest indicator. For each item, participants selected one of three responses: ‘not true’, ‘somewhat true’, or ‘definitely true’.

- *Have other people ever read your mind?*
- *Have you had messages sent to you through TV or radio?*
- *Have you ever felt that you were under control of some special power?*
- *Have you ever heard voices or sounds that no-one else can hear?*
- *Have you ever seen things that other people could not see?*
- *Have you ever felt that you have extra-special powers?*
- *Have you ever thought that people are following you or spying on you?*

Total scores were calculated by assigning yes, 1 point, maybe, 0.5 points and no, 0 points which were summed for each of the seven questions and those with a score higher than 2 were categorized as the PE group, in line with the pilot APSS validation study (Kelleher et al., 2011).

#### Predictor variables

**Emotional and behavioral difficulties.** Strengths and Difficulties Questionnaire (SDQ) measured emotional and behavioral difficulties, a widely used behavioral screening tool suitable for adolescents aged 11–16 (Goodman, Meltzer, & Bailey, 1998). Consisting of 25 statements about the participant’s behavior in the last 6 months, the instrument measures 5 subscales each with 5 questions that examine emotion, personal conduct, hyperactivity/inattention, peer problems, and pro-social behavior (Goodman, 2001). The >16 total cutoff was selected in this study due to its reported validity for identifying clinically significant difficulties and its alignment with prior research investigating associations between mental health and PEs (Rønning, Handegaard, Sourander, & Mørch, 2004). This approach ensures consistency with previous studies in Irish and international populations, particularly those addressing clinical significance rather than broader population norms. This variability supports the use of the SDQ not only as a predictor but also in combination with other psychosocial exposures to examine whether associations with PEs varied across predefined psychosocial groups.

**Mental wellbeing.** The Short Warwick-Edinburgh Mental Wellbeing Scale (SWEMWBS), a 7-item self-report instrument was developed to assess positive mental health in the general population and evaluate the impact of policies or interventions aimed at improving wellbeing (Tennant et al., 2007). Participants were asked to reflect on their experiences over the past 2 weeks, rating each statement on a 5-point Likert scale (None of the time, Rarely, Some of the time, Often, All of the time). Items included:

- *I’ve been feeling optimistic about the future*
- *I’ve been feeling useful*
- *I’ve been feeling relaxed*
- *I’ve been dealing with problems well*
- *I’ve been thinking clearly*
- *I’ve been feeling close to other people*
- *I’ve been able to make my mind up about most things*

Scoring adhered to procedures outlined by the University of Warwick (Tennant et al., 2007). In line with previous research, high and low scorers were identified using population-based cutoffs (Ng Fat, Scholes, Boniface, Mindell, & Stewart-Brown, 2017) with the bottom 15% of scores (≤19.5) indicating low mental wellbeing. For analysis, SWEMWBS scores were recoded into a binary variable: scores ≤19.5 were classified as low mental wellbeing, while scores above this threshold served as the reference group.

**Demographics.** Ethnicity was coded into three categories as per survey: White Irish (reference), Other White, and Black, Asian, and Other Minority group. Gender was self-reported using four response options: male (reference), female, non-binary/transgender, and prefer not to disclose. Age was approximated using self-reported year of birth. This variable was used descriptively to characterize the sample. Region was based on the location of participants’ schools. Responses were initially grouped as North Dublin, Cavan, and Monaghan. For analysis, Cavan and Monaghan were combined to represent a predominantly rural region, while North Dublin was retained as a predominantly urban region. Financial hardship was assessed with the item: ‘My parents/carers hardly have enough money to pay for necessities (e.g., food, housing)’, rated on a 5-point Likert scale (Almost never to Almost always). Responses of ‘Often’ or ‘Almost always’ were categorized as financial hardship; all other responses were coded as no financial hardship.

**Adverse life events**. Participants self-reported adverse life events, which included serious accident, severe illness, parental separation or divorce, serious arguments with parents or carers, witnessing serious arguments, witnessing physical violence at home, witnessing psychological violence or abuse at home, involvement in physical violence at home, the death of a parent, carer, or sibling, the death of a friend, breakup with a girlfriend or boyfriend, rejection by friends, separation from a friend, receiving an exceptionally low grade, parental job loss, parental imprisonment, parental substance use problems, dismissal from class or being sent to the principal, and expulsion from school.

Recent exposure was assessed by identifying whether participants experienced any adverse life events during the past 30 days, reflecting acute stressors. Cumulative exposure was captured as the total number of adverse life events reported over the past 12 months, excluding those occurring in the most recent 30 days, to represent the chronic burden.

#### Protective factors

Parental support was assessed using items measuring how easily participants could receive care, warmth, and advice from their parents or guardians. This variable reflects the availability of supportive family relationships and was tested both as a direct protective factor and as a potential buffer against the impact of adverse life events.

Adult support at school was measured through items assessing whether participants felt cared for, noticed, and supported by adults in their school environment. This construct was included to evaluate the protective role of school-based relationships in adolescent mental health, as well as their buffering potential.

Perceived safety was assessed through participants’ reported sense of safety at home and at school. These were analyzed separately to explore their roles as protective factors, particularly in mitigating the association between adversity and PEs.

### Data cleaning

Surveys with less than 85% completion were excluded to ensure sufficient data for analysis. Core demographic items were positioned early in the questionnaire to maximize response rates. Missingness increased in later sections, particularly for PEs and some psychosocial predictors, consistent with response fatigue. For the PE outcome, the missingness was low (2.8%). Patterns of missingness were examined across gender identity, ethnicity, and SDQ status, and there was no evidence that minority groups were disproportionately more likely to have incomplete surveys. Given the low proportion of missing data (<5%) and absence of substantial differential missingness, complete-case analysis without multiple imputation was used for multivariable models.

### Statistical analysis

Descriptive statistics were calculated for all demographic and predictor variables among participants with ≥85% completion. Included and excluded participants were compared to assess potential bias. A multivariable logistic regression model was used to examine associations between psychosocial predictors and PEs. Risk and protective factors were selected based on prior theory.

First, exploratory unadjusted analyses were undertaken for all candidate psychosocial, demographic, and contextual variables to assess their associations with PE and to inform subsequent model specification. These analyses were not intended for standalone inference but guided iterative model development, including evaluation of alternative specifications for stability and interpretability.

Second, a main-effects multivariable logistic regression model was fitted to estimate average adjusted associations between each risk factor and PE, including the main effect of gender identity, ethnicity, feeling of safety, and other covariates.

Third, based on a priori hypotheses regarding subgroup heterogeneity, a final model was fitted using jointly defined categorical exposure terms (e.g. SDQ × gender, recent adversity × parental support, mental wellbeing × adult support). This parameterization estimates subgroup-specific a*OR*s relative to predefined reference categories within a single regression framework, allowing direct comparison of joint exposure groups without fitting separate stratified models. Such joint exposure specification is commonly used in epidemiological analyses examining heterogeneity across social categories.

Gender identity was specified a priori as a key grouping variable for examining associations between SDQ scores and PEs, informed by minority stress frameworks suggesting that gender-diverse adolescents may experience distinct psychosocial stressors. However, the dataset did not include direct measures of discrimination or enacted stigma.

Sensitivity analyses additionally examined alternative joint exposure specifications by ethnic minority status using SDQ-by-ethnicity categories. When modelled simultaneously with SDQ × gender joint exposure categories, the SDQ × ethnicity terms were weaker and did not materially alter interpretation; therefore, only SDQ × gender combinations were retained in the final model.

Recent adverse life events (past 30 days) were entered as binary; cumulative events (past 12 months, excluding recent) were entered as continuous. Other predictors were coded as binary or categorical using predefined thresholds. Demographic covariates included gender identity (male [reference], female, prefer not to disclose and non-binary/transgender), ethnicity (White Irish, Other White, and Black, Asian, and Other Minority group), region, and financial hardship. Adjusted odds ratios (aORs) with 95% confidence intervals were reported, and robust standard errors were applied.

### Assumption testing

The assumptions of multivariable logistic regression were assessed to ensure model validity. Multicollinearity was evaluated using variance inflation factors (VIFs), which ranged from 1.00 to 1.33 (mean VIF = 1.14), indicating no significant collinearity among predictors. The relationship between the continuous predictor, negative life events over the past 12 months (excluding the past 30 days), and the log odds of the outcome was confirmed to be linear. The assumption of independence was addressed by applying robust standard errors to account for potential clustering or heteroscedasticity in the data. The model specification was evaluated using the linktest, which indicated a significant result for the squared predicted value (_hatsq). However, as the study utilized secondary data with a pre-specified theoretical framework, no further adjustments to the model were made. Other diagnostics, including assessments of multicollinearity, linearity, and influential observations, confirmed the robustness of the model.

## Results

### Sample demographics

A total of 4,404 survey entries were recorded. After applying the >85% survey completion inclusion criterion, the final analytical sample consisted of 4,005 participants, which was used for all analyses (Table 1). Among the sample, 2,015 (50.3%) participants identified as male and 1,816 (45.3%) as female, while 111 (2.8%) identified as non-binary or transgender. Gender identity was not disclosed by 61 (1.5%). A total of 2,823 (70.5%) identified as White Irish, 728 (18.2%) as Other White, while 449 (11.2%) identified as Black, Asian, or other minority ethnicity. Five participants (0.1%) did not report their ethnicity.

**Table 1.** PE study sample demographics

Demographics	Total n (%)	Non-PE groupn (%)	PE group n (%)	Missing n (%)
**Gender**	**4,005 (100%)**	**2,699 (67.4%)**	**1,193 (29.8%)**	**113 (2.8%)**
Male	2,015 (50.3%)	1,414 (32.3%)	535 (13.4%)	66 (1.7%)
Female	1,816 (45.3%)	1,203 (30.0%)	571 (14.3%)	42 (1.1%)
Non-binary/transgender	111 (2.8%)	52 (1.3%)	55 (1.4%)	1 (0.0%)
Not disclosed	61 (1.5%)	29 (0.7%)	31 (0.8%)	4 (0.1%)
**Ethnicity**	**4,005 (100%)**	**2,699 (67.4%)**	**1,193 (29.8%)**	**113 (2.8%)**
White Irish	2,823 (70.5%)	1,988 (49.6%)	770 (19.2%)	65 (1.6%)
Other White	728 (18.2%)	254 (6.3%)	174 (4.3%)	21 (0.5%)
Black, Asian, or other minority ethnicity	449 (11.2%)	254 (6.3%)	174 (4.3%)	21 (0.5%)
Not disclosed	5 (0.1%)	3 (0.07%)	1 (0.02%)	1 (0.02%)
**Age (years)**	**4,005 (100%)**	**2,699 (67.4%)**	**1,193 (29.8%)**	**113 (2.8%)**
≤14	468 (11.7%)	267 (6.7%)	167 (4.2%)	34 (0.8%)
15	1,511 (37.7%)	994 (24.8%)	469 (11.7%)	48 (1.2%)
16	1,486 (37.1%)	1,023 (25.5%)	425 (10.6%)	38 (0.9%)
17	466 (11.6%)	466 (11.6%)	103 (2.6%)	12 (0.3%)
≥18	73 (1.8%)	45 (1.1%)	25 (0.6%)	3 (0.08%)
Not disclosed	1 (0.02%)	1 (0.02%)	0 (0.0%)	0 (0.0%)
**Region**	**4,005 (100%)**	**2,699 (67.4%)**	**1,193 (29.8%)**	**113 (2.8%)**
Predominantly rural (Cavan/Monaghan)	1,565 (39.1%)	1,142 (28.5%)	382 (9.5%)	41 (1.0%)
Predominantly urban (North Dublin)	2,440 (60.9%)	1,558 (39.0%)	811 (20.2%)	72 (1.8%)
**Financial hardship**	**4,005 (100%)**	**2,699 (67.4%)**	**1,193 (29.8%)**	**113 (2.8%)**
No financial hardship	3,606 (90.0%)	2,457 (61.4%)	1,049 (26.2%)	100 (2.5%)
Frequent financial hardship	358 (8.9%)	213 (5.3%)	132 (3.3%)	13 (0.3%)
Not disclosed	41 (1.0%)	29 (0.7%)	12 (0.3%)	0 (0.0%)

Participants were categorized into age groups based on self-reported birth year. A total of 468 participants (11.7%) were 14 years or younger, 1,511 (37.7%) were 15 years old.; 1,486 participants (37.1%) aged 16, and 466 participants (11.6%) aged 17. The remaining 73 participants (1.8%) were 18 years or older, while one participant (0.02%) did not report their age.

Furthermore, participants were categorized based on the location of their school. A total of 1,565 participants (39.1%) were from Cavan/Monaghan, while 2,440 participants (60.9%) were from North Dublin. Among the PE group, 382 participants (9.5%) were from Cavan/Monaghan, and 811 participants (20.3%) were from North Dublin.

A total of 358 participants (8.94%) reported frequent financial hardship, defined as parents/carers struggling to afford necessities often or always. The majority, 3,606 (90.0%), reported no financial hardship, while 41 (1.02%) did not provide financial information.

PEs were calculated for 3,889 individuals (M = .32, SD = 1.50) based on responses to seven screening items, allowing at most one missing value per respondent. Using the established threshold, 1,193 (29.8%) had scores ≥2, classifying them in the PE group, while 2,699 (67.4%) scored below 2 not meeting the threshold for PEs. Data were missing for 113 participants (2.8%), who were excluded from relevant analyses.

### Multivariable logistic regression modeling

In line with the analytic plan, a main-effects multivariable logistic regression model was first fitted. A second model was fitted using joint exposure parameterization defined a priori combining key predictors (SDQ × gender, recent adversity × parental support, mental wellbeing × adult support). This approach estimates subgroup-specific adjusted odds ratios relative to predefined reference categories within a single regression framework. The joint exposure model demonstrated slightly improved explanatory value and is presented as the primary inferential model.

**Figure 1.**
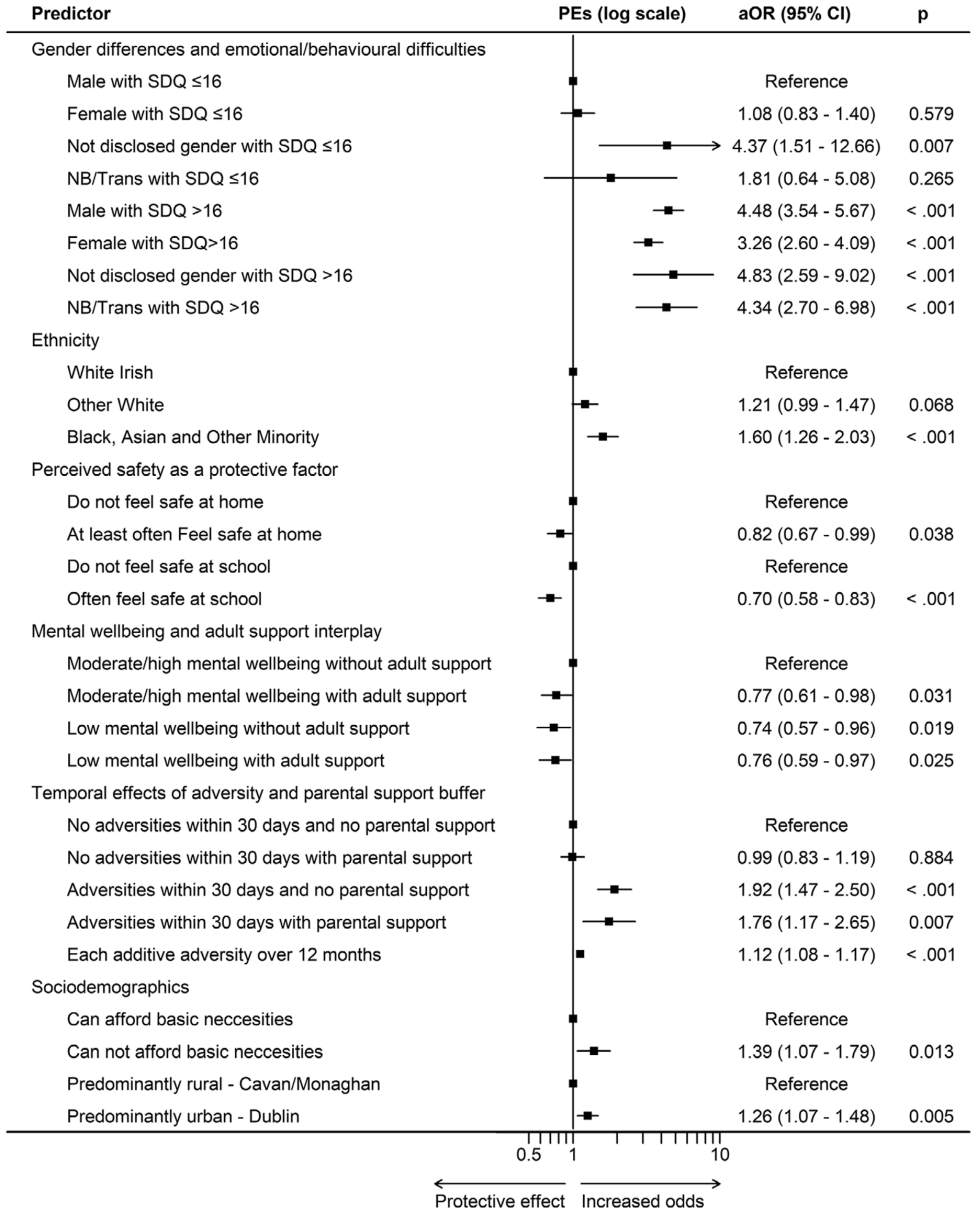
Forest plot of adjusted OR (95% CI) from main-effect multivariable logistic regression for key predictors of PEs.

**Figure 2.**
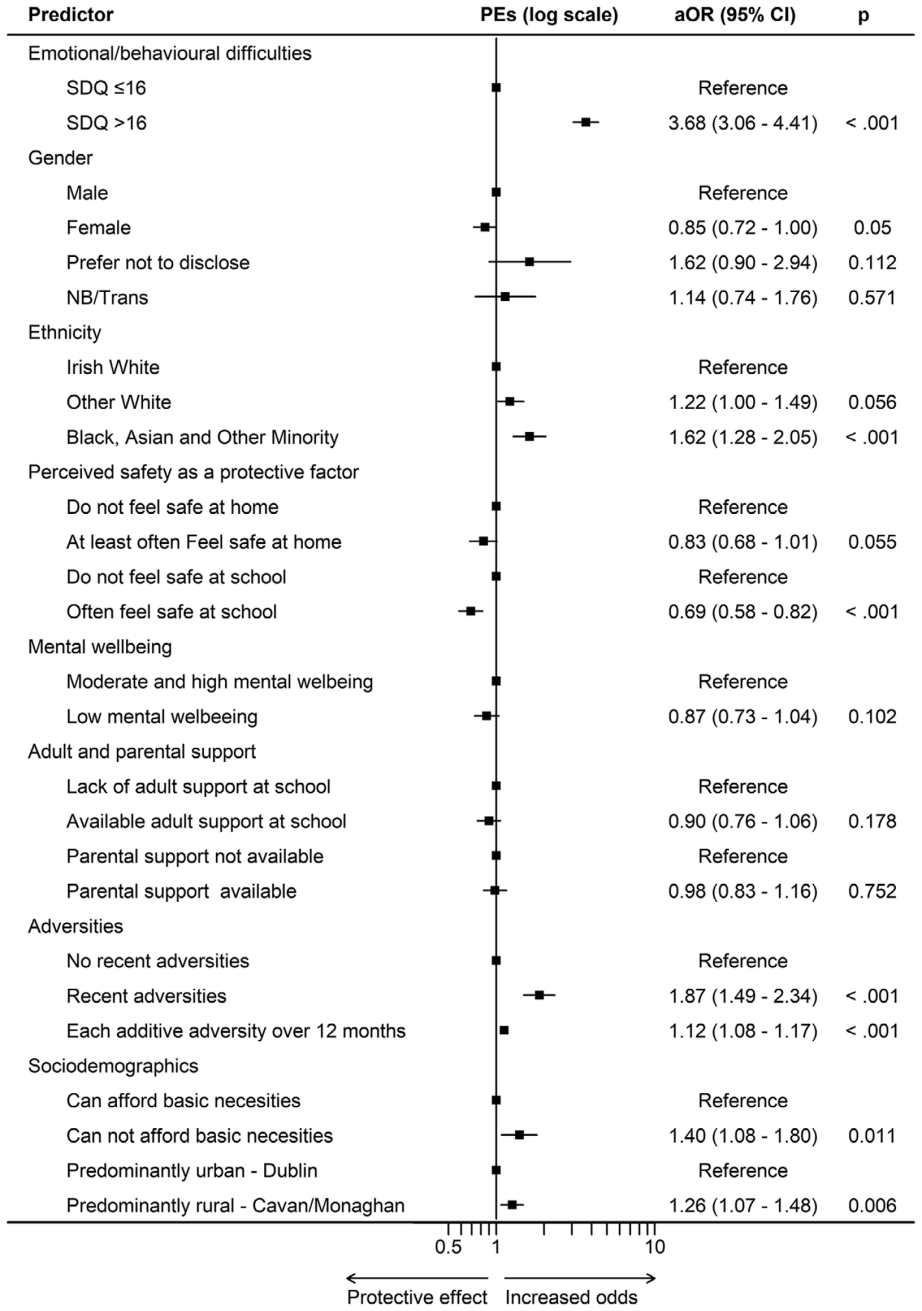
Forest plot of adjusted OR (95% CI) from the joint exposure multivariable logistic regression model for key predictors of PEs.

The main effect model included 3,786 participants and was statistically significant (Wald χ^2^(15) = 539.01, *p* < .001; pseudo R^2^ = 0.1328). The final model incorporated prespecified joint exposure categories defined by cross-classified predictors. The logistic regression model was statistically significant (Wald χ^2^(20) = 542.04, *p* < .001; pseudo R^2^ = 0.1355). Model fit indices indicated moderate explanatory power. A total of 3,728 participants were included in the analysis (Figures 1 and 2).

#### Gender differences and emotional/behavioural difficulties on PE

In the main-effects model, high emotional/behavioural difficulties (SDQ > 16; reference ≤16) were associated with higher odds of PEs (a*OR* = 3.67, 95% CI 3.06–4.40, *p* < .001). Within gender effect (reference: males), females had lower odds of PEs (a*OR* = 0.85, 95% CI 0.72–1.00, *p* = .050), while non-binary/transgender participants and those preferring not to disclose gender did not differ significantly.

In the final model, compared to male adolescents with an SDQ ≤ 16 (reference group), elevated odds of PEs were observed for participants with SDQ > 16 across all gender groups. Among male adolescents, high SDQ scores were associated with higher odds of PEs (a*OR* 4.47, 95% CI 3.53–5.67, *p* < .001). Female adolescents with SDQ > 16 had a*OR* 3.25 (95% CI 2.59–4.08, *p* < .001), those preferring not to disclose gender (a*OR* 4.83, 95% CI 2.58–9.02, *p* < .001), and non-binary/transgender participants (a*OR* 4.33, 95% CI 2.69–6.97, p < .001). Among participants scoring below the SDQ threshold, those preferring not to disclose gender had higher odds relative to the reference group (a*OR* 4.36, 95% CI 1.50–12.66, *p* = .007), whereas females and non-binary/transgender participants did not differ significantly. The SDQ × gender joint exposure categories were jointly statistically significant (Wald χ^2^(7) = 217.23, *p* < .001), supporting inclusion of the specification in the final model.

#### Perceived safety as a protective factor

In the main-effects model, perceived school safety (reference: not safe at school) was associated with lower odds of PEs (a*OR* = 0.68, 95% CI 0.57–0.82, *p* < .001). Home safety was not statistically significant as compared to the lack of perceived safety at home.

In the final model including jointly defined exposure categories, perceived safety remained protective with adolescents who reported feeling safe at school had lower odds of being in the PE group compared with those who did not (a*OR* = 0.69, 95% CI 0.58–0.83, *p* < .001). Similarly, perceived safety at home was associated with lower odds of PEs (a*OR* = 0.81, 95% CI 0.66–0.99, *p* = .038) compared with those who did not feel safe at home.

#### Mental wellbeing and adult support interplay

Compared to adolescents with low mental wellbeing and low adult school support (reference group), those with low wellbeing but high school support had lower PE odds (a*OR* = 0.77, 95% CI 0.61–0.98, *p* = .031). Similarly, adolescents with high mental wellbeing but low school support exhibited 27% lower odds (a*OR* = 0.73, 95% CI 0.57–0.95, *p* = .008). The combination of higher mental wellbeing and adult support was similarly associated with reduced odds (a*OR* 0.75, 95% CI 0.58–0.96, *p* = .025).

#### Temporal effects of adverse life events and parental support buffer

In the main effects model, recent adverse life events (reference: none in past 30 days) were associated with increased odds (a*OR =* 1.86, 95% CI 1.49–2.34, *p* < .001). Cumulative adverse life events over the prior 12 months (continuous) were positively associated with PEs (a*OR* per event a*OR* = 1.12, 95% CI 1.07–1.17, *p* < .001).

In the final model, differences were observed across joint exposure categories of recent adversity (within 30 days) and parental support. Compared to adolescents with no recent adversity and no parental support, those who experienced recent adversity but had no parental support had significantly higher PE odds (a*OR* = 1.91, 95% CI 1.47–2.49, *p* = .011). Among those with high parental support, recent adversity remained associated with elevated odds of PEs compared with those with no recent adversity and high parental support, although the magnitude of association was modestly attenuated (a*OR* = 1.76, 95% CI 1.17–2.65, *p* < .001).

#### Sociodemographic predictors of odds

In the main effects model, ethnicity was parameterized with White Irish as the reference category. Black, Asian, and Other Minority group participants had higher odds (*aOR* = 1.61, 95% CI 1.27–2.05, *p* < .001), whereas Other White participants did not differ statistically.

Compared to White Irish adolescents (reference group), those from a Black, Asian, and Other Minority group had much higher odds of PEs (a*OR* = 1.59, 95% CI 1.26–2.02, *p* < .001), while Other White participants did not differ significantly. Compared to adolescents who reported no financial hardship (reference group), those experiencing financial hardship had 38% higher odds of being in the PE group (*aOR* = 1.38, 95% CI 1.07–1.79, *p* = .013). Compared to adolescents living in Cavan/Monaghan (reference group), those living in North Dublin had higher PE odds (*aOR* = 1.24, 95% CI 1.07–1.48, *p* = .005).

## Discussion

This study aimed to explore the prevalence and associated factors of PEs among adolescents residing in predominantly urban North Dublin, and predominantly rural Cavan and Monaghan in 2021. Our findings provide insights into the complex interplay of demographic, socioeconomic, mental health, and protective factors that contribute to PEs in this population. The results underscore the importance of understanding the multifaceted nature of adolescent mental health, particularly in the context of stigma and psychosocial stressors affecting minoritized groups.

### Gender related emotional/behavioral difficulties and the risk of PEs

Elevated emotional and behavioral difficulties were associated with substantially increased odds of PEs across all gender categories. However, the magnitude of association differed. Adolescents with SDQ >16 who identified as male had more than fourfold increased odds relative to the reference group (SDQ ≤16, male). The corresponding OR among females was smaller, whereas the ORs for non-binary/transgender adolescents and those preferring not to disclose gender were of similar or greater magnitude than that observed for males. These findings indicate heterogeneity in effect size across gender categories, rather than a uniform pattern of amplification. The SDQ-associated increase in odds was larger among males than females, suggesting that high levels of emotional and behavioral difficulties may translate into risk of PEs more sharply for males in this cohort. This pattern contrasts with assumptions that female adolescents uniformly exhibit greater vulnerability to PEs and highlights the importance of modelling psychosocial burden and gender jointly rather than as independent main effects.

Alongside male–female heterogeneity, the joint estimates also differentiated gender-diverse participants from those who preferred not to disclose gender. Non-binary/transgender adolescents showed markedly elevated odds of PEs in the context of high SDQ difficulty scores, consistent with the possibility that psychosocial burden and gender-related stressors may compound risk. In contrast, adolescents who preferred not to disclose gender exhibited elevated odds even at lower SDQ scores, suggesting vulnerability that may not be captured by measured emotional/behavioral difficulties alone. One interpretation is that non-disclosure could reflect concerns about stigma, safety, or concealment stress; however, this remains speculative in the absence of direct measures of discrimination or reasons for non-disclosure.

### Perceived safety as protective factor

Perceived environmental safety was independently associated with lower odds of PEs in the fully adjusted model including joint exposure categories. Adolescents reporting that school felt safe showed substantially reduced odds, with a more modest but statistically significant association also observed for home safety. These findings are consistent with prior work linking perceived contextual security to adolescent mental health outcomes (Robustelli, Newberry, Whisman, & Mittal, 2017). While the cross-sectional nature of the data precludes causal inference, the pattern suggests that environments perceived as safe may mitigate vulnerability to PEs during adolescence.

### Mental wellbeing and adult support interplay

The joint modelling of mental wellbeing and adult support indicated that adolescents reporting both low wellbeing and no adult support exhibited the highest odds of PEs. Relative to this group, the presence of adult support alone and higher mental wellbeing alone were each associated with reduced odds. The combined presence of higher wellbeing and adult support did not demonstrate a markedly greater reduction than either factor independently, suggesting limited evidence for a synergistic buffering effect.

These findings indicate that both individual wellbeing and perceived adult support are relevant, but their joint pattern does not suggest a strong offsetting effect whereby adult support fully compensates for the risk associated with low wellbeing. It remains possible that unmeasured supports or contextual factors contribute to variation across these groups.

### Temporal effect of adversity and parental support buffer

The findings indicate that parental support plays a role in mitigating the psychological impact of recent adversity (within the past 30 days). Recent adversity was associated with elevated odds of PEs irrespective of parental support. However, the magnitude of association was modestly attenuated in the presence of parental support (a*OR* 1.76 vs 1.91), suggesting partial buffering. Despite this attenuation, risk remained substantially elevated, indicating that parental support may mitigate but does not eliminate the impact of acute adversity.

However, even with support, recent adversity remained a substantial risk factor, highlighting the resilience limits of this buffer under acute stress conditions.

In contrast, cumulative adversity over the past 12 months (excluding the most recent 30 days) demonstrated a compounding effect, with each additional adverse event associated with a 12% increase in the odds of PEs. This highlights the enduring influence of chronic stress on adolescent mental health, independent of short-term support mechanisms. These findings build on previous research by Kelleher et al. (2013), which highlighted the critical impact of adverse experiences on adolescent mental health.

### Ethnicity

Adolescents from Black, Asian, and Other Minority backgrounds exhibited significantly higher odds of PEs compared with White Irish adolescents, whereas no significant difference was observed for Other White participants. These associations persisted in the fully-adjusted multivariable model, suggesting that ethnic disparities were not fully explained by measured psychosocial risk factors.

Ireland’s student population has become increasingly diverse over the past 15 years, yet relatively little is known about the everyday school experiences of ethnic minority adolescents, particularly in relation to peer dynamics and teacher relationships (Ní Dhuinn & Keane, 2023). Although the present study did not directly assess experiences of discrimination, stigma, or exclusion, the elevated odds observed in minority ethnic groups are consistent with literature linking minority ethnicity to adverse mental health outcomes.

These findings underscore the importance of culturally responsive mental health services and school environments that actively promote safety, inclusion, and belonging for racially and ethnically minoritized adolescents.

### Heterogeneity in psychosocial risk across social groups

The findings suggest that psychosocial risk factors for PEs do not operate uniformly across adolescents. Emotional and behavioral difficulties were strongly associated with PEs across all groups, but the magnitude of association varied by gender category. Additionally, adolescents who preferred not to disclose gender showed elevated risk independent of measured symptom burden, and ethnic disparities persisted after adjustment for psychosocial factors.

These patterns underscore the importance of modelling subgroup heterogeneity rather than assuming uniform effects. While minority stress frameworks may offer one explanatory lens, direct measures of discrimination and social adversity were not included, limiting mechanistic interpretation. Future research should integrate structural and interpersonal stress measures to clarify how social context shapes the emergence of PEs.

### Limitations and future directions

This study has several limitations, including its cross-sectional design, which precludes causal inference. While we identified associations between multiple factors and PEs, the underlying mechanisms remain unclear and warrant further investigation. A study examining the lived experience of adolescents in Ireland, including those from marginalized backgrounds, may offer deeper insights into how psychosocial factors influence PE and help-seeking behaviors. We have purposely referred to minoritized groups rather than ‘minorities’ to reflect structural exclusion, as limitations in the demographic categories collected in this survey restrict the analysis of certain identities. To better understand the impact of marginalization and adversity on PEs, future research must ensure inclusive demographic data collection. Data were collected during the COVID-19 pandemic, a period of heightened psychological stress, disrupted routines, and limited social support for many adolescents. While this context may have influenced rates of PEs and psychosocial factors, the cross-sectional design precludes attributing any observed effects specifically to pandemic-related conditions.

The dataset is based on anonymized survey responses and does not include respondent identifiers or session linkage. As a result, it is not possible to verify whether each entry corresponds to a unique individual or to distinguish between complete and partial submissions. While the survey was administered in supervised classroom settings, some records contained limited item completion, suggesting early termination of the questionnaire. Consequently, the reported sample reflects the number of survey entries rather than a confirmed count of individual participants, and student-level participation rates could not be directly established.

## References

[r1] Brokmeier, L. L., Firth, J., Vancampfort, D., Smith, L., Deenik, J., Rosenbaum, S., … Schuch, F. B. (2020). Does physical activity reduce the risk of psychosis? A systematic review and meta-analysis of prospective studies. Psychiatry Research, 284, 112675. 10.1016/j.psychres.2019.112675. 31757637

[r2] Crush, E., Arseneault, L., Moffitt, T. E., Danese, A., Caspi, A., Jaffee, S. R., … Fisher, H. L. (2018). Protective factors for psychotic experiences amongst adolescents exposed to multiple forms of victimization. Journal of Psychiatric Research, 104, 32–38. 10.1016/j.jpsychires.2018.06.011. 29929082 PMC6109202

[r3] Dolphin, L., Dooley, B., & Fitzgerald, A. (2015). Prevalence and correlates of psychotic like experiences in a nationally representative community sample of adolescents in Ireland. Schizophrenia Research, 169(1–3), 241–247. 10.1016/j.schres.2015.09.005. 26416443

[r4] Flentje, A., Heck, N. C., Brennan, J. M., & Meyer, I. H. (2020). The relationship between minority stress and biological outcomes: A systematic review. Journal of Behavioral Medicine, 43(5), 673–694. 10.1007/s10865-019-00120-6. 31863268 PMC7430236

[r5] Goodman, R. (2001). Psychometric properties of the strengths and difficulties questionnaire. Journal of the American Academy of Child & Adolescent Psychiatry, 40(11), 1337–1345. 10.1097/00004583-200111000-00015. 11699809

[r6] Goodman, R., Meltzer, H., & Bailey, V. (1998). The strengths and difficulties questionnaire: A pilot study on the validity of the self-report version. European Child & Adolescent Psychiatry, 7(3), 125–130. 10.1007/s007870050057. 9826298

[r7] Hanevik, H., Hestad, K. A., Lien, L., Teglbjaerg, H. S., & Danbolt, L. J. (2013). Expressive art therapy for psychosis: A multiple case study. The Arts in Psychotherapy, 40(3), 312–321. 10.1016/j.aip.2013.05.011.

[r8] Healy, C., Campbell, D., Coughlan, H., Clarke, M., Kelleher, I., & Cannon, M. (2018). Childhood psychotic experiences are associated with poorer global functioning throughout adolescence and into early adulthood. Acta Psychiatrica Scandinavica, 138(1), 26–34. 10.1111/acps.12907. 29855047

[r9] Kelleher, I., & Cannon, M. (2012). The authors reply: All that shines is not psychosis … but is still clinically important. Psychological Medicine, 42(8), 1788–1790. 10.1017/S0033291712001250.

[r10] Kelleher, I., Connor, D., Clarke, M. C., Devlin, N., Harley, M., & Cannon, M. (2012). Prevalence of psychotic symptoms in childhood and adolescence: A systematic review and meta-analysis of population-based studies. Psychological Medicine, 42(9), 1857–1863. 10.1017/s0033291711002960. 22225730

[r11] Kelleher, I., Harley, M., Murtagh, A., & Cannon, M. (2011). Are screening instruments valid for psychotic-like experiences? a validation study of screening questions for psychotic-like experiences using in-depth clinical interview. Schizophrenia Bulletin, 37(2), 362–369. 10.1093/schbul/sbp057. 19542527 PMC3044617

[r12] Kelleher, I., Keeley, H., Corcoran, P., Ramsay, H., Wasserman, C., Carli, V., … Cannon, M. (2013). Childhood trauma and psychosis in a prospective cohort study: Cause, effect, and directionality. American Journal of Psychiatry, 170(7), 734–741. 10.1176/appi.ajp.2012.12091169. 23599019

[r13] Mendo-Lázaro, S., León-Del-Barco, B., Polo-Del-Río, M. I., Yuste-Tosina, R., & López-Ramos, V. M. (2019). The Role of Parental Acceptance⁻Rejection in emotional Instability during adolescence. International Journal of Environmental Research and Public Health, 16(7). 10.3390/ijerph16071194. PMC6480184 30987100

[r14] Meyer, I. H. (2003). Prejudice, social stress, and mental health in lesbian, gay, and bisexual populations: Conceptual issues and research evidence. Psychological Bulletin, 129(5), 674–697. 10.1037/0033-2909.129.5.674. 12956539 PMC2072932

[r15] Meyer, I. H., Russell, S. T., Hammack, P. L., Frost, D. M., & Wilson, B. D. M. (2021). Minority stress, distress, and suicide attempts in three cohorts of sexual minority adults: A U.S. probability sample. PLoS One, 16(3), e0246827. 10.1371/journal.pone.0246827. 33657122 PMC7928455

[r16] Meyrose, A., Klasen, F., Otto, C., Gniewosz, G., Lampert, T., & Ravens-Sieberer, U. (2018). Benefits of maternal education for mental health trajectories across childhood and adolescence. Social Science & Medicine, 202, 170–178. 10.1016/j.socscimed.2018.02.026. 29554584

[r17] Ng Fat, L., Scholes, S., Boniface, S., Mindell, J., & Stewart-Brown, S. (2017). Evaluating and establishing national norms for mental wellbeing using the short Warwick-Edinburgh mental well-being scale (SWEMWBS): Findings from the health survey for England. Quality of Life Research, 26(5), 1129–1144. 10.1007/s11136-016-1454-8. 27853963 PMC5376387

[r18] Ní Dhuinn, M., & Keane, E. (2023). But you don’t look Irish’: Identity constructions of minority ethnic students as ‘non-Irish’ and deficient learners at school in ireland. International Studies in Sociology of Education, 1–30. 10.1080/09620214.2021.1927144.

[r19] Robustelli, B. L., Newberry, R. E., Whisman, M. A., & Mittal, V. A. (2017). Social relationships in young adults at ultra high risk for psychosis. Psychiatry Research, 247, 345–351. 10.1016/j.psychres.2016.12.008. 27987484 PMC5217827

[r20] Rønning, J. A., Handegaard, B. H., Sourander, A., & Mørch, W. T. (2004). The strengths and difficulties self-report questionnaire as a screening instrument in Norwegian community samples. European Child & Adolescent Psychiatry, 13(2), 73–82. 10.1007/s00787-004-0356-4. 15103532

[r21] Staines, L., Healy, C., Kelleher, I., Cotter, D., Burns, A., & Cannon, M. (2023). The association between transient childhood psychotic experiences and psychosocial outcomes in young adulthood: Examining the role of mental disorders and adult attachment. Early Intervention in Psychiatry. 10.1111/eip.13382. PMC10947326 36646439

[r22] Stainton, A., Chisholm, K., Woodall, T., Hallett, D., Reniers, R., Lin, A., & Wood, S. J. (2021). Gender differences in the experience of psychotic-like experiences and their associated factors: A study of adolescents from the general population. Schizophrenia Research, 228, 410–416. 10.1016/j.schres.2021.01.008. 33556674

[r23] Tennant, R., Hiller, L., Fishwick, R., Platt, S., Joseph, S., Weich, S., … Stewart-Brown, S. (2007). The Warwick-Edinburgh mental well-being scale (WEMWBS): development and UK validation. Health and Quality of Life Outcomes, 5(1), 63. 10.1186/1477-7525-5-63. 18042300 PMC2222612

[r24] Vargas, T., Rakhshan Rouhakhtar, P. J., Schiffman, J., Zou, D. S., Rydland, K. J., & Mittal, V. A. (2020). Neighborhood crime, socioeconomic status, and suspiciousness in adolescents and young adults at Clinical High Risk (CHR) for psychosis. Schizophrenia Research, 215, 74–80. 10.1016/j.schres.2019.11.024. 31759810 PMC7036021

